# Event and Comment

**Published:** 1914-09-15

**Authors:** 


### DENTAL REGISTER.

**Vol. LXVIII. September 15, 1914. No. 9**

### EVENT AND COMMENT.

### SOMETHING NEW IN DENTAL LEGISLATION.

We quote below from an editorial in the *Northwestern Journal of Dentistry*, published in Portland, Oregon, a proposition by a notorious advertising dentist to amend the state law regulating practice according to his own notions. We haven't an idea that such suicidal laws could ever be enacted in any state, but it shows what absurd notions a selfish man will entertain. This man has plenty of money and threatens to use it generously to accomplish his purpose. This same man got his start by street faking from a gilded band wagon in one of our largest eastern cities, where he has a large number of advertising offices. He is now living in California and aims to do for the Pacific Coast what he did for the Atlantic. It is to be hoped the people of the west may not be hoodwinked by his sophistries. Because they are so daringly absurd, there is a possibility that the profession may not see the necessity for organized opposition until it is too late. If a definite quietus can be put on this proposition in Oregon it will be helpful in all the other states.

"In the Oregonian of June 28th there appeard a quarter page advertisement under the caption, 'I will wipe out the Ethical Dental Trust.' After a false and misleading preamble four amendments to the dental laws of this state were prepared, each one followed by a most vicious and distorted explanation cleverly designed to fool the voters.

MEN OF THE DENTAL PROFESSION, READ THESE AMENDMENTS CAREFULLY AND DIGEST

THEM UNDERSTANDINGLY! DO YOU REALIZE WHAT THEY MEAN?

1—*That it shall be unlawful for any dentist to use cocaine or arsenic, or any solution containing either or both of these poisons, in the practice of dentistry in this state.*

"This law will stop the Ethical Dental Trust from poisoning the people, and leaving in many patients a craving for cocaine.

"2—*That a competent dental nurse shall be present as attendant at all times in every operating room during each operation.*

"This law will be a protection to every woman patient. No woman will be compelled to be alone in a room with a dentist during operations, as is often the case at present because the Ethical Dental Trust will not pay a living wage to nurses.

"3—*That within six months after the passage of this law every person practicing dentistry in the state shall be compelled to pass an examination in theory and practice before a special state board of dental examiners composed of advertising as well as non-advertising dentists; and further, that any dentist in the State of Oregon who is a member of any society, association or organization which has a scale of fees or price list of charges shall be denied the right to take this examination, and any dentist thereafter who holds membership in any such society, association or organization shall be forever barred from practicing dentistry in the State of Oregon.*

This law will weed out a lot of incompetent dentists who have been given licenses to practice by state boards under the control of the Ethical Dental Trust in years past, and also prevent the trust from maintaining its combine in restraint of trade.

"4—*That the State Board of Dental Examiners shall be compelled to admit to practice in this state any dentist who holds a legal certificate to practice dentistry in any other state of the Union, providing that said state will likewise accept the certificates issued by the state of Oregon.*

"This law will leave the state dental board free to enforce the law without fear or favor for the public good and take it out of the control of any clique or combine. It is the principle of reciprocity in dentistry, giving the public full protection and every dentist an even break before the law.

"I am opposed to all trusts and especially a dental trust. I believe that when the people of this state understand what is being done by this Ethical Dental Trust under the cover of laws intended for the public good, they will demand a new deal and a square deal.

"I am the equal in skill, training and experience of any member of the Ethical Dental Trust, and I don't propose to be branded a quack simply because I will not join the trust in plundering the public when it is suffering with the toothache.

"I am in favor of laws protecting the public against incompetent and dishonest dentists, and I believe the people of Oregon are opposed to laws being made in the interest of a dental trust."

